# Cardiac resynchronisation therapy in paediatric and congenital heart disease: differential effects in various anatomical and functional substrates

**DOI:** 10.1136/hrt.2008.160465

**Published:** 2009-03-22

**Authors:** J Janoušek, R A Gebauer, H Abdul-Khaliq, M Turner, L Kornyei, O Grollmuß, E Rosenthal, E Villain, A Früh, T Paul, N A Blom, J-M Happonen, U Bauersfeld, J R Jacobsen, F van den Heuvel, T Delhaas, J Papagiannis, C Trigo, for the Working Group for Cardiac Dysrhythmias and Electrophysiology of the Association for European Paediatric Cardiology

**Affiliations:** 1 Department of Paediatric Cardiology, University of Leipzig, Heart Centre, Leipzig, Germany; 2 Kardiocentrum and Cardiovascular Research Centre, University Hospital Motol, Prague, Czech Republic; 3 German Heart Centre Berlin, Berlin, Germany; 4 Bristol Royal Infirmary and University of Bristol, Bristol, UK; 5 Hungarian Paediatric Heart Centre, Budapest, Hungary; 6 Evelina Children’s Hospital, Guy’s and St Thomas’ Trust, London, UK; 7 Département de Cardiologie Pédiatrique, Hôpital Necker, Paris, France; 8 Paediatric Cardiology Unit, Rikshospitalet University Hospital, Oslo, Norway; 9 Department of Paediatric Cardiology and Intensive Care, University Hospital, Göttingen, Germany; 10 Department of Paediatric Cardiology, Leiden University Medical Centre, Leiden, The Netherlands; 11 Division of Paediatric Cardiology, Department of Paediatrics, Helsinki University Central Hospital, Helsinki, Finland; 12 Division of Paediatric Cardiology, University Children’s Hospital of Zurich, Zurich, Switzerland; 13 Department of Paediatrics, Rigshospitalet, Copenhagen, Denmark; 14 Beatrix Children’s Hospital, Division of Paediatric Cardiology, University Medical Centre Groningen, The Netherlands; 15 Division of Paediatric Cardiology, AZ Maastricht, Maastricht, The Netherlands; 16 Department of Paediatric Cardiology, Onassis Cardiac Surgery Centre, Athens, Greece; 17 Servico de Cardiologia Pediatrica, Hospital de Santa Marta, Lisboa, Portugal

## Abstract

**Background:**

Cardiac resynchronisation therapy (CRT) is increasingly used in children in a variety of anatomical and pathophysiological conditions, but published data are scarce.

**Objective:**

To record current practice and results of CRT in paediatric and congenital heart disease.

**Design:**

Retrospective multicentre European survey.

**Setting:**

Paediatric cardiology and cardiac surgery centres.

**Patients:**

One hundred and nine patients aged 0.24–73.8 (median 16.9) years with structural congenital heart disease (n = 87), congenital atrioventricular block (n = 12) and dilated cardiomyopathy (n = 10) with systemic left (n = 69), right (n = 36) or single (n = 4) ventricular dysfunction and ventricular dyssynchrony during sinus rhythm (n = 25) or associated with pacing (n = 84).

**Interventions:**

CRT for a median period of 7.5 months (concurrent cardiac surgery in 16/109).

**Main outcome measures:**

Functional improvement and echocardiographic change in systemic ventricular function.

**Results:**

The z score of the systemic ventricular end-diastolic dimension decreased by median 1.1 (p<0.001). Ejection fraction (EF) or fractional area of change increased by a mean (SD) of 11.5 (14.3)% (p<0.001) and New York Heart Association (NYHA) class improved by median 1.0 grade (p<0.001). Non-response to CRT (18.5%) was multivariably predicted by the presence of primary dilated cardiomyopathy (p = 0.002) and poor NYHA class (p = 0.003). Presence of a systemic left ventricle was the strongest multivariable predictor of improvement in EF/fractional area of change (p<0.001). Results were independent of the number of patients treated in each contributing centre.

**Conclusion:**

Heart failure associated with ventricular pacing is the largest indication for CRT in paediatric and congenital heart disease. CRT efficacy varies widely with the underlying anatomical and pathophysiological substrate.

Cardiac resynchronisation therapy (CRT) has become a standard treatment for adults with idiopathic or ischaemic cardiomyopathy and systemic ventricular dyssynchrony. Improvement in ventricular function, functional status and decreased mortality has been shown in recent large randomised trials.^1^ ^2^ CRT has also been applied to the paediatric and congenital heart disease population. So far only a few case reports,^3^^–^5 small uncontrolled studies^6^^–^9 and one retrospective multicentre survey^10^ have reported benefit from CRT in this complex population. The aim of this multicentre European experience was to record the current practice of this evolving therapy, indications and predictors of benefit or failure in various anatomical and pathophysiological situations.

## METHODS

### Data collection

Data from patients with structural congenital heart disease and congenital complete atrioventricular block regardless of age and from patients with dilated cardiomyopathy under the age of 21 years subjected to CRT up to the deadline of data collection were gathered retrospectively. Data collected consisted of demographics, underlying heart disease and its surgical treatment, morphology of the systemic ventricle, preceding ventricular pacing, New York Heart Association (NYHA) class and the method used to identify electromechanical dyssynchrony. Details of CRT system implantation were documented. Heart transplant listing, complications of CRT as well as death were noted. Non-responders were defined as those not responding clinically (decrease in NYHA class) and having no improvement in systemic ventricular function. Given the retrospective study design and the wide range of ages, functional assessment using the 6 min walk test or quantitative cardiopulmonary testing was not required.

### Echocardiographic data

End-diastolic and end-systolic dimensions of the systemic ventricle were measured at the point of peak diastolic systemic ventricular free wall outward motion and peak systolic inward motion, respectively, and expressed as a z score indexed to a normal systemic left ventricle (LV).^11^ Systemic ventricular shortening fraction was calculated according to the following formula: ((end-diastolic dimension – end-systolic dimension)/end-diastolic dimension) × 100. In patients with a systemic LV the ejection fraction (EF) was measured by either the biplane Simpson method or the Teichholz method. Care was taken to use the same measurement method before CRT and at the last follow-up in each individual patient. For a systemic right ventricle (RV) or functionally single ventricle, the fractional area of change was measured from the apical four-chamber view. The grade of systemic atrioventricular valve was assessed using usual semiquantitative grading as none = 0, mild = 1, moderate = 2 and severe  = 3.^12^ No details of mechanical dyssynchrony evaluation other than the method used were obtained.

### Statistical analysis

**Figure 1:**
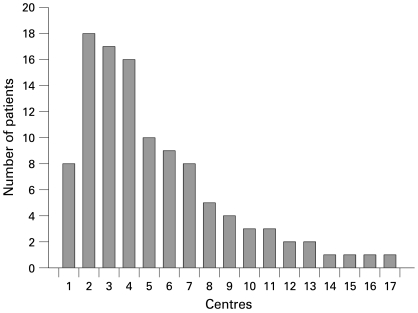
Number of patients contributed by each centre (centres are numbered according to the number used in the author affiliations).

Values are expressed as either median (range) or mean (SD) depending on the distribution pattern of the data. Differences in continuous variables among groups of patients were evaluated by one-way analysis of variance, two-tailed *t* test or Mann–Whitney rank sum test, as appropriate. Paired comparisons were performed by paired *t* test. Predictors of improvement in systolic function and non-response to CRT were evaluated by univariate tests and were entered into a multivariable analysis (backward stepwise regression) if showing significant univariate correlation. The following independent variables were used: age at CRT, type of heart disease, systemic ventricular morphology, preceding ventricular pacing, initial QRS duration, QRS shortening by CRT, initial NYHA class, z score of the systemic ventricular end-diastolic diameter, shortening fraction, EF or fractional area of change and grade of systemic atrioventricular valve regurgitation. For the comparison between large- and small-volume centres the institutions were divided into two groups with either >10 or ⩽10 reported patients (fig 1). p Values <0.05 were considered significant. Calculations were performed using SigmaStat 3.1 and SigmaPlot 9.1 for Windows (SPSS, Chicago, Illinois, USA).

## RESULTS

### Demographics

The study group comprised 109 patients from 17 European paediatric cardiology and cardiac surgery centres (fig 1) with a median age 16.9 years (range 2.9 months–73.8 years) at initiation of CRT, and subsequent follow-up of a median of 7.5 months. The systemic ventricle was morphologically left in 69 patients, right in 36 and four patients had a functionally single ventricle. Eighty-two patients (75.2%) had a history of preceding major cardiac surgery (tables 1 and 2). Twenty-three patients from two centres (No 2 (Kardiocentrum and Cardiovascular Research Centre, Czech Republic) and 6 (Evelina Children’s Hospital, Guy’s and St Thomas’ Trust, London)) have been included in the previously published study by Dubin *et al*.^10^ Follow-up for these patients is longer in the current report.

**Table 1:** Patient data and CRT effects according to diagnostic group

Patient data	All (n = 109)	CHD (CRT only) (n = 71)	CHD (CRT + concurrent cardiac surgery) (n = 16)	Congenital AV block (n = 12)	DCMP (n = 10)	p Value*
Age at CRT (years), median	16.9	25.5	13.9	3.6	12.7	<0.001
Follow-up on CRT (months), median	7.5	8.8	4.0	9.5	2.3	0.099
Initial QRS (ms), median	160	160	160	155	144	0.082
Initial SVEDD (z score), median	3.3	2.5	2.6	3.7	5.5	0.131
Initial EF/FAC (%), median	27.0	27.5	24.5	24.5	22.0	0.602
Initial SAVV regurgitation (grade), median	1	1	0	1	2	0.589
Initial NYHA class (median)	2.5	2.5	2.0	2.5	4.0	0.058
Change in QRS (ms), median	−40§	−40§	−46§	−40§	−15	0.268
Change in SVEDD (z score), median	−1.1§	−1.2§	−0.8	−2.2¶	−0.4	0.582
Change in EF/FAC (%), mean (SD)	+11.5 (14.3)§	+9.9 (13.8)§	+12.3 (17.1)¶	+23.5 (12.2)¶	+10.8 (11.9)%	0.358
Change in SAVV regurgitation (grade), median	−1§	0	0	−1¶	0	0.556
Change in NYHA class (median)	−1.0§	−1.0§	−1.0†	−1.0‡	0.0	0.028
Non-responders	15/94	7/60	0/13	2/12	6/9	<0.001

*Significance levels across diagnostic categories; significance levels inside diagnostic categories pre-CRT vs post-CRT: †p<0.01; ‡p<0.005; §p<0.001; ¶p<0.05.

AV, atrioventricular; CHD, congenital heart disease; CRT, cardiac resynchronisation therapy; DCMP, dilated cardiomyopathy; EF, ejection fraction; FAC, fractional area of change; NS, non-significant; NYHA, New York Heart Association; SAVV, systemic atrioventricular valve; SVEDD, systemic ventricular end-diastolic dimension.

**Table 2:** Structural cardiac diagnoses

Systemic LV (n = 69)		Systemic RV (n = 36)		Functionally single ventricle (n = 4)	
Diagnosis	n	Diagnosis	n	Diagnosis	n
TOF	11	cCTGA	20	SV	2
VSD	10	d-TGA	12	HLHS	1
d-TGA	4	DORV	4	Unbalanced AVSD	1
AS	3				
ASD	4				
AVSD	3				
COA	2				
PA-VSD	2				
Shone complex	2				
Other	6				
Normal heart	22				

AS, aortic stenosis; ASD, atrial septal defect; AVSD, atrioventricular septal defect; cCTGA, congenitally corrected transposition of great arteries; COA, coarctation of aorta; DORV, double outlet right ventricle; d-TGA, d-transposition of great arteries; HLHS, hypoplastic left heart syndrome; LV, left ventricle; PA-VSD, pulmonary atresia with ventricular septal defect; RV, right ventricle; SV, single ventricle; TOF, tetralogy of Fallot.

### Electrical dyssynchrony

Twenty-five patients (23%) had ventricular dyssynchrony during intrinsic conduction. Ten patients (9%) had left bundle branch block with a systemic LV and five patients had right bundle branch block with a systemic right or functionally single ventricle. The remaining 10/25 patients had non-specific QRS prolongation. The majority of study patients (84/109, 77%) had dyssynchrony associated with single-site ventricular pacing for a median of 5.6 years (range 3 days–25.1 years) before the upgrade to CRT. Of those, all but six patients were paced from the subpulmonary chamber.

### Mechanical dyssynchrony

The majority of patients (105/109, 96%) underwent evaluation of mechanical dyssynchrony by either conventional echocardiography (92 patients) and/or by tissue Doppler imaging (67 patients) without a prespecified protocol.

### CRT implantation

A transvenous approach was used in 45 (41%) patients, thoracotomy in 36 (33%) patients and a combined approach in 28 (26%) patients. Patients in the transvenous group were significantly older: median 33.5 (7.1–73.8) vs 8.4 (0.2–67.5) years (p<0.001). Two ventricular leads were used in 105 patients. Two patients with a systemic LV and RV, respectively, had resynchronisation by a single ventricular lead placed transvenously or by thoracotomy at the site of ventricular conduction delay. Finally, one patient with transposition of great arteries after the atrial switch procedure and another patient with a functionally single ventricle received three ventricular leads in an attempt to better resynchronise a severely dilated ventricle. Additional concurrent cardiac surgery was performed in 16 patients (15%). CRT systems with defibrillating capability were used in 20 patients (18%).

### QRS change

QRS duration decreased from a median of 160 ms before CRT implantation to 130 ms at last follow-up (p<0.001). Initial QRS duration was significantly longer in patients paced before CRT (mean (SD) 169 (28) ms) than in non-paced patients (mean 141 (30) ms, p<0.001). QRS shortening was also more pronounced (median −40 vs −10 ms, p = 0.001) in the previously paced group.

### Reverse ventricular remodelling

Sufficient echocardiographic data to allow analysis was available in 89/109 patients. Table 1 summarises the impact of CRT on the total population and the four different diagnostic groups, with a significantly higher proportion of non-responders in the dilated cardiomyopathy group. For all further analysis of change in systemic ventricular size and function after CRT, patients with concurrent cardiac surgical procedures were excluded. After doing so the calculated overall increase in systemic ventricular EF/fractional area of change and decrease in the z score of the systemic ventricular end-diastolic dimension still remained highly significant with +11.2 (13.7)% and median −1.3, respectively (p<0.001 for both).

Table 3 summarises the differences between patients with systemic LV and RV. Initial systemic ventricular end-diastolic dimension and its decrease after CRT were significantly greater in patients with systemic LV. Patients with a systemic LV who were upgraded to CRT from previous ventricular pacing had a better response than the rest of the population (mean increase in EF/fractional area of change of +14.0 (16.5) vs 7.7 (10.9)%, p = 0.101, median decrease in the z score of systemic ventricular end-diastolic dimension of −2.1 vs −0.8, p = 0.036 and decrease in median NYHA class from 3.0 to 1.0 as compared with no change in median values, p = 0.030). Approach to ventricular lead placement (transvenous vs thoracotomy/combined) as well as patient volume (large vs small volume centres) had no significant influence on improvement of EF/fractional area of change (+13.8 (13.5)% vs 7.6 (13.6)% and +9.1 (15.3)% vs 14.8 (12.4)%, respectively, p value NS for both). Predictors of increase in EF/fractional area of change could be evaluated in 31/109 patients with available data and follow-up >3 months identifying the presence of a systemic LV (p<0.001) and higher initial grade of systemic AV valve regurgitation (p = 0.002) as the only significant factors in a multivariable analysis.

**Table 3:** Patients’ data and CRT effects according to systemic ventricular anatomy (patients with CRT and concurrent cardiac surgical procedure excluded)

Patients’ data	Systemic LV (n = 62)	Systemic RV (n = 27)	p Value*
Age at CRT (years), median	13.3	28.8	0.002
Follow-up on CRT (months), median	8.6	7.3	0.965
Initial QRS (ms), median	160	160	0.722
Initial SVEDD (z score), median	4.7	2.1	0.002
Initial EF/FAC (%), mean (SD)	30.6 (15.8)	28.8 (10.0)	0.723
Initial SAVV regurgitation (grade) (median)	1	2	0.025
Initial NYHA class (median)	3.0	2.0	0.215
Change in QRS (ms), median	−40§	−21§	0.877
Change in SVEDD (z score), median	−2.1§	−0.5	0.039
Change in EF/FAC (%), mean (SD)	+13.3 (14.7)§	+7.2 (9.9)¶	0.195
Change in SAVV regurgitation (grade), median	−1§	−1¶	0.600
Change in NYHA class (median)	−1.0§	−1.0‡	0.380
Non-responders	11/54	3/22	0.745

*Significance levels across diagnostic categories; significance levels inside diagnostic categories pre- vs post-CRT: ‡p<0.005; §p<0.001; ¶p<0.05.

CRT, cardiac resynchronisation therapy; EF, ejection fraction; FAC, fractional area of change; LV, left ventricle; NS, non-significant; NYHA, New York Heart Association; RV, right ventricle; SAVV, systemic atrioventricular valve; SVEDD, systemic ventricular end-diastolic dimension.

### Systemic atrioventricular valve regurgitation

Patients with concurrent cardiac surgical procedures were excluded from this analysis. Systemic mitral valve regurgitation decreased from a median grade 1 to 0 after CRT (p<0.001) and systemic tricuspid valve insufficiency from a median grade 2 to 1 (p = 0.037). Both initial and latest follow-up systemic atrioventricular valve regurgitation grades were significantly higher in patients with systemic RV than in those with systemic LV (p = 0.025 and 0.005, respectively, table 3). The decrease in systemic AV valve regurgitation was significantly correlated with the increase in EF/fractional area of change (R = 0.536, p<0.001).

### Functional improvement

After excluding patients with concurrent cardiac surgical procedures the overall NYHA class improved by a median of 1.0 grade (p<0.001). Table 1 gives data for specific patients. No change in NYHA class could be seen in patients with dilated cardiomyopathy. There was a positive correlation between initial NYHA class and systemic ventricular end-diastolic diameter (R = 0.554, p<0.001) but no correlation with shortening fraction or EF/fractional area of change.

### Functionally single ventricle

Table 4 summarises the data for patients with a functionally single ventricle. Three of the four patients improved after CRT.

**Table 4:** Patients with functionally single ventricle

Patient No	Age (years)	Length of FUP on CRT (months)	Type of surgical repair	Diagnosis	Previous ventricular pacing	No of ventricular leads	NYHA class before CRT	NYHA class after CRT	Outcome
57	9.5	3.5	TCPC	SV-LV	Yes	2	3.0	3.0	Non-responder
59	3.7	0.5	TCPC	HLHS	No	3	3.0	2.0	Improved
70	11.0	7.9	BCPA	Unbalanced AVSD	Yes	2	2.0	1.0	Improved
79	30.3	19.9	TCPC	SV	Yes	2	2.5	2.0	Improved

AVSD, atrioventricular septal defect; BCPA, bi-directional cavopulmonary anastomosis; CRT, cardiac resynchronisation therapy; FUP, follow-up; HLHS, hypoplastic left heart syndrome; NYHA, New York Heart Association; SV, unspecified single ventricle; SV-LV, morphologically left single ventricle; TCPC, total cavopulmonary connection.

### Patients who underwent concurrent cardiac surgical procedures

Sixteen of the 109 patients (15%) had major cardiac surgery along with CRT implementation, most commonly systemic atrioventricular valve replacement or repair (7/16) in the presence of systemic left (n = 3) or right ventricle (n = 4) or pulmonary artery banding after previous atrial switch for transposition of great arteries (n = 2). In general, these patients had significant improvement in NYHA class (median −1.0 grade, p = 0.008) and EF/fractional area of change (+12.3 (17.1)%, p = 0.031) similar to the rest of the population (table 1). None of these patients died or was judged a non-responder.

### CRT complications and discontinuation

Acute complications were noted in 10 patients (9.2%): pocket haematoma (four patients), CRT lead dislodgement (two patients, both were transvenous implants under the age of 15 years) and pneumothorax, ventricular fibrillation, fever or rise in pacing threshold during anaesthesia leading to resuscitation (one patient each). CRT had to be discontinued during follow-up in nine patients (8.3%) because of lead failure in six, worsening of heart failure in two and infection of the pacing system in one patient. There was no significant difference in acute complications and CRT discontinuation between patients with transvenous and thoracotomy/mixed lead systems (4.7 vs 15.6% and 10.9 vs 4.4%, respectively) and between large and small volume centres (9.8 vs 8.6% and 11.8 vs 5.2%, respectively); p value NS for all.

### Non-responders

Fifteen of 81 patients with available data and without concurrent cardiac surgery (18.5%) were identified as non-responders (table 5). Risk factors for non-response were evaluated by univariate and multivariable analysis with the only independent multivariable predictors being primary dilated cardiomyopathy and initial poor NYHA class (table 6). Of the six non-responders with primary dilated cardiomyopathy one had a polyglucosan storage defect and two of the remaining five with no apparent aetiology had baseline QRS duration <100 ms, indicating absence of electrical dyssynchrony. These two patients showed an increase in QRS duration after CRT. Although CRT indication was based on echocardiographic evaluation of mechanical dyssynchrony the interpretation may have been wrong and improvement was not achieved. There was no difference in the proportion of thoracotomy or mixed lead systems between the responder and non-responder group (33/66 vs 8/15 patients). Also there was no patient volume-dependent effect (14.3% of non-responders in the large volume vs 20.8% in the small volume centres, p = 0.560).

**Table 5:** Non-responders and non-survivors

No	Age (years)	Length of FUP (months)	Diagnostic group	Systemic ventricle*	SVEDD* (z score)	EF/FAC* (%)	SF* (%)	Pacing before CRT	NYHA class*	Non-responder	Outcome
11	3.6	3.6	CHD	RV	5.5	30.0	13.0	Yes	2.0	Yes	Alive, HTx performed
14	41.1	15.1	CHD	RV	N/A	N/A	N/A	Yes	3.0	Yes	Alive, HTx listed
27	11.3	8.6	CHD	LV	8.5	41.0	16.0	Yes	3.0	Yes	Alive
28	2.4	5.8	CCAVB	LV	3.3	21.0	9.0	Yes	2.0	Yes	Alive
29	5.3	0.5	DCMP	LV	6.6	20.0	9.0	No	4.0	Yes	Died, end-stage HF
31	16.9	2.3	DCMP	LV	5.5	24.0	11.0	Yes	3.0	Yes	Died, malignant VT
36	52.5	7.3	CHD	RV	0.3	13.9	21.0	Yes	3.0	Yes	Alive, HTx listed
57	9.5	3.5	CHD	SV-LV	7.1	23.0	13.0	Yes	3.0	Yes	Alive
62	25.8	1.8	CHD	LV	6.9	N/A	21.0	Yes	4.0	Yes	Died, malignant VT
63	20.9	4.1	DCMP	LV	6.1	N/A	11.0	No	4.0	Yes	Alive, HTx performed
71	1.7	1.3	DCMP	LV	13.1	7.0	2.0	No	4.0	Yes	Alive, HTx listed
85	4.9	0.1	CCAVB	LV	10.3	N/A	6.5	Yes	4.0	Yes	Died, end-stage HF
91	3.4	4.5	CHD	LV	1.9	N/A	N/A	Yes	4.0	No	Died, unknown aetiology
92	11.2	1.0	DCMP	LV	4.2	N/A	17.0	No	4.0	Yes	Died, end-stage HF
93	2.5	0.0	DCMP	LV	8.6	N/A	15.0	No	4.0	Yes	Alive, worsening of HF after CRT, HTx listed
94	61.3	4.3	CHD	LV	2.5	N/A	N/A	No	3.0	Yes	Died, endocarditis, end-stage HF

*Before CRT.

CCAVB, congenital complete atrioventricular block; CHD, congenital heart disease; CRT, cardiac resynchronisation therapy; DCMP, dilated cardiomyopathy; EF, ejection fraction; FAC, fractional area of change; FUP, follow-up; HF, heart failure; HTx, heart transplantation; LV, left ventricle; N/A, not available; NYHA, New York Heart Association; RV, right ventricle; SF, shortening fraction; SVEDD, systemic ventricular end-diastolic dimension; SV-LV, morphologically left single ventricle; VT, ventricular tachycardia.

**Table 6:** Predictors of non-response to cardiac resynchronisation therapy (patients with CRT and concurrent cardiac surgical procedure excluded)

Predictors	Responders (n = 66)	Non-responders (n = 15)	p Value	
			Univariate	Multivariable
Congenital heart disease (%)	80.3	46.7	0.018	NS
Congenital complete AV block (%)	15.2	13.3	1.000	Not tested
Dilated cardiomyopathy (%)	4.5	40	<0.001	0.002
Initial NYHA class (median)	2.0	3.0	<0.001	0.003
Age at CRT (years), median	18.1	11.2	0.447	Not tested
Systemic right ventricle (%)	28.8	20.0	0.749	Not tested
Pacing before CRT (%)	81.8	60.0	0.087	Not tested
Initial SVEDD (z score), mean (SD)	+3.5 (2.7)	+6.3 (3.4)	0.003	NS
Initial SF (%), mean (SD)	17.3 (9.9)	12.9 (4.2)	0.148	Not tested
Initial EF/FAC (%), mean (SD)	31.6 (14.5)	22.5 (10.2)	0.101	Not tested
Initial SAVV regurgitation (grade), median	1	2	0.682	Not tested
Initial QRS duration (ms), median	160	150	0.301	Not tested
QRS shortening by CRT (ms), median	−40	−30	0.357	Not tested
Thoracotomy or mixed lead system (%)	50.0	53.3	1.000	Not tested

AV, atrioventricular; CRT, cardiac resynchronisation therapy; EF, ejection fraction; FAC, fractional area of change; NYHA, New York Heart Association; SAVV, systemic atrioventricular valve; SF, shortening fraction; SVEDD, systemic ventricular end-diastolic dimension.

It is notable that the non-responders included four patients (Nos 27, 28, 62 and 85, table 5) with the otherwise favourable combination of a systemic LV and pacing-associated dyssynchrony. These four patients had severely dilated LVs (z score of the systemic ventricular end-diastolic diameter: +3.3 to +10.3) and a high NYHA class. Patient No 27 underwent a Rastelli procedure with subsequent mitral valve replacement and the myocardial dysfunction may have been a consequence of multiple operations. Patient No 28 had isolated congenital complete atrioventricular block and no apparent reason for the lack of response.

Patient No 62 died of malignant ventricular tachycardia 2 months after upgrade to biventricular pacing. Finally, patient No 85 with end-stage heart failure died just 2 days after upgrade to CRT owing to unstable haemodynamics.

### Negative treatment outcomes

A total of 22 patients had negative outcomes owing to non-response followed eventually by heart failure-related death or heart transplantation (n = 15, table 5) or owing to discontinuation of CRT for technical reasons or infection (n = 7).

### Heart transplantation

Four of the 10 patients originally listed for heart transplantation were removed from the transplant list because of improvement in cardiac function after CRT. No significant predictors of improvement could be found in this small group.

### Death

During follow-up seven patients died (table 5). These patients were similar to the non-responders characterised by a higher NYHA class (median 4.0 as compared with 2.0 in the survivors, p<0.001). Four of the patients died because of end-stage heart failure. Two of the seven died of ventricular arrhythmia.

## DISCUSSION

CRT is a standard treatment option for adults with left ventricular failure associated with mechanical dyssynchrony. The benefits of CRT in paediatric patients and patients with congenital heart disease have previously only been documented in one large study including 103 patients.^10^ This retrospective multicentre survey is the first analysis of CRT effects in paediatric patients and patients with congenital heart disease based on European data. As compared with the study by Dubin *et al*^10^ our paper defines for the first time risk factors for non-response or lack of improvement in systolic function and presents additional important information about the use of CRT in specific patient groups.

### Patient population

This study reports patients with different pathophysiology of systemic ventricular desynchronisation when compared with adult idiopathic and ischaemic cardiomyopathy. The proportion of patients with the typical combination of systemic LV and left bundle branch block was low (9%). Furthermore, a majority had ventricular desynchronisation associated with pacing (77%). This figure exceeds the previously published 45% by Dubin *et al*^10^ and suggests that alternative pacing strategies should be considered in this patient population. Such finding is consistent with the published adult and paediatric data on the adverse effects of right ventricular pacing on LV synchrony,^13^ histology,^14^ function^7^ ^8^ ^15^ ^16^ and on morbidity and mortality due to heart failure.^17^^–^19

### Systemic ventricular morphology

A systemic LV was predictive of improvement in systolic function and showed more extensive reverse remodelling, whereas systemic right ventricular patients did worse. Both pre- and post-CRT systemic atrioventricular valve regurgitation was higher in subjects with systemic RV. This may be one of the reasons for the less successful reverse remodelling in this group. Patients with a systemic RV may mostly have structural tricuspid valve regurgitation potentially less improvable by resynchronisation than functional mitral regurgitation in systemic LV.^20^ This is underscored by the fact that overall improvement in systolic function did significantly correlate with the decrease in systemic AV valve regurgitation. Thus the combination of CRT with surgery aimed at decreasing tricuspid valve regurgitation may be a valuable strategy as reflected by our subgroup with concurrent cardiac surgical procedures, and described elsewhere.^7^ Other reasons for the smaller benefit of CRT in a systemic RV may lie in the different right ventricular architecture and decreased myocardial perfusion reserve as described in patients after atrial switch repair.^21^ ^22^ Dubin *et al*^10^ reported similar improvement of EF in patients with a systemic RV as in the whole CRT population, but did not analyse the change in systemic ventricular size or systemic atrioventricular valve regurgitation or compare these patients specifically with the systemic LV cohort.

### Ventricular dyssynchrony associated with pacing

Correction of electrical dyssynchrony by CRT was highly successful in this patient subgroup, as shown by the significant decrease in QRS duration in this and a previous study.^10^ Patients with a systemic LV upgraded to CRT demonstrated major clinical improvement and reverse LV remodelling. Dubin *et al*^10^ could not find a difference in EF improvement between patients upgraded to CRT from single-site pacing and those with primary CRT. In their study, however, patients were not separated according to systemic ventricular morphology. As seen in our non-responder group some paced patients with a severely dilated LV may not benefit from an upgrade to CRT. CRT might potentially have prevented irreversible LV damage if applied earlier in the course of the disease. Recently, improvement of LV function has been described after a simple change in pacing site from the right ventricular epicardium to the LV apex.^23^ It remains to be seen whether such a strategy may constitute an alternative to upgrading to biventricular pacing.

### Non-responders

A total of 15 non-responders (18.5% of patients with available data and no concurrent cardiac surgery) were defined by the participating investigators. This compares favourably with an around one-third non-response rate in most adult CRT series and is slightly higher than the previously published 10.7% in a similar population.^10^

Two independent predictors of non-response were identified. Patients with idiopathic dilated cardiomyopathy responded poorly and less well than reported in the adult CRT population, where resynchronisation was more successful in this setting than with ischaemic aetiology. Several factors may have been responsible including different aetiology of dilated cardiomyopathy in the young (metabolic disease was present in one of the non-responders) and potential absence of mechanical dyssynchrony in another two, whose baseline QRS duration was short and increased after CRT. The limited follow-up in this group was rather a consequence than a reason for lack of response. Table 5 shows that four of the six non-responders in the group with dilated cardiomyopathy either died or received a transplant shortly after CRT implementation. Worsening of haemodynamics before a later positive effect would be quite unusual as contraction efficacy improves immediately after CRT. Thus CRT could not reverse the malignant course of the disease.

The other risk factor for non-response was poor initial NYHA class. Whether this finding would argue in favour of offering CRT at an earlier stage to prevent irreversible ventricular deterioration is unclear from the present data. Whereas in the report by Dubin *et al*^10^ the only difference between responders and non-responders was the higher initial systemic EF in the latter (“too well to benefit” as speculated by the authors themselves) and their analysis did not include type of heart disease as an independent variable, our study was able to define risk factors for non-response. In contrast to the cited report our definition of non-responders required, besides the absence of an increase in EF, lack of improvement in functional NYHA class. Thus criteria used for identification of non-responders were stronger and this may itself account for some differences in the analysis.

### Influence of patient volume

Interestingly, this study showed that CRT could be equally safe and effective independently of the patient volume treated in each contributing centre. All the participating institutions were, however, tertiary centres combining an extensive cardiological and surgical experience in treating congenital heart disease. Thus, institutional background rather than the actual volume of patients treated is probably more important to achieve satisfactory results.

### Limitations

The limitations of this study are its retrospective design, lack of predefined indication criteria for CRT implantation, relatively soft criteria for identification of CRT response (functional and/or echocardiographic improvement), incomplete clinical and echocardiographic datasets, methodological difficulties in the evaluation of a systemic right ventricle and lack of other functional assessment than the NYHA classification. The systemic ventricular shortening fraction used for quantification of systolic function may be inaccurate in the presence of ventricular dyssynchrony. Its use was, however, limited to evaluating changes due to CRT in selected individual patients in whom EF or fractional area of change were not available. The shortening fraction was not used for any analysis of overall CRT efficacy. Further, accuracy of NYHA classification is questionable in the congenital atrioventricular block group owing to the median age of 3.6 years. Given the age of the remaining patients, the small size of the congenital atrioventricular block group and the limitation of the alternative Ross heart failure score (applicable only to infants), the authors nevertheless found that NYHA was useful and practical for describing functional CRT efficacy in this retrospective setting.

Patients with primary dilated cardiomyopathy seem to be a mixed population in this study making general conclusions about CRT efficacy difficult. And, finally, this study was not primarily focused on use of CRT in heart transplant candidates and although some patients could be delisted after CRT, others were transplanted because of non-response. Thus the utility of CRT in this patient group cannot be clearly assessed from our data. Despite these limitations and a wide variety of heart disease included, the study group was large enough to allow for meaningful and significant conclusions.

## CONCLUSION

Overall, CRT in this population was at least as effective as in adults with heart failure. The response was, however, dependent on the structural and pathophysiological substrate being most favourable after upgrades from single site to biventricular pacing in patients with a systemic LV and generally less favourable in patients with a systemic RV and in children with primary dilated cardiomyopathy, where proper patient selection might improve the results. NYHA class was poorly correlated with systemic ventricular function and it is questionable whether it is a major criterion for CRT indication in congenital heart disease.
